# Supplementary material for: Influenza A Virus Infection Reactivates Human Endogenous Retroviruses Associated with Modulation of Antiviral Immunity
Source: Viruses. 2022 Jul 21;14(7):1591. doi: 10.3390/v14071591 (PMC9320126; doi:10.3390/v14071591)
Supplement: Supplementary file 1 [file viruses-14-01591-s001.zip › MotifAnalysis.zip!/MotifOutput_SC35MdNS1/homerResults/motif13.similar.html]

motif13

## Information for motif13

A
G
T
C
C
G
T
A
A
G
C
T
A
G
T
C
A
G
T
C
C
T
A
G
C
G
T
A
A
C
T
G
A
C
T
G
C
G
T
A
C
G
T
A
A
C
G
T
  
Reverse Opposite:  

C
G
T
A
A
C
G
T
A
C
G
T
A
G
T
C
A
G
T
C
A
C
G
T
A
G
T
C
A
C
T
G
A
C
T
G
C
T
G
A
A
C
G
T
A
C
T
G
  

|  |  |
| --- | --- |
| p-value: | 1e-17 |
| log p-value: | -4.086e+01 |
| Information Content per bp: | 1.949 |
| Number of Target Sequences with motif | 6.0 |
| Percentage of Target Sequences with motif | 7.79% |
| Number of Background Sequences with motif | 2.1 |
| Percentage of Background Sequences with motif | 0.00% |
| Average Position of motif in Targets | 108.5 +/- 47.5bp |
| Average Position of motif in Background | 159.2 +/- 8.9bp |
| Strand Bias (log2 ratio + to - strand density) | 10.0 |
| Multiplicity (# of sites on avg that occur together) | 1.00 |
| Motif File: | file (matrix) reverse opposite |

### Similar de novo motifs found

|  |  |  |  |  |  |  |  |
| --- | --- | --- | --- | --- | --- | --- | --- |
| Rank | Match Score | Redundant Motif | P-value | log P-value | % of Targets | % of Background | Motif file |
| 1 | 0.617 | A G T C A G T C A T G C C G T A A C T G A C T G C G T A C G A T A C G T A G T C | 1e-10 | -24.269635 | 7.79% | 0.07% | motif file (matrix) |
| 2 | 0.636 | A G T C A T G C C G T A A C T G A C T G C G T A C G A T C G A T | 1e-3 | -8.634936 | 7.79% | 1.06% | motif file (matrix) |
